# Reputation Effects in Social Networks Do Not Promote Cooperation: An Experimental Test of the Raub & Weesie Model

**DOI:** 10.1371/journal.pone.0155703

**Published:** 2016-07-01

**Authors:** Rense Corten, Stephanie Rosenkranz, Vincent Buskens, Karen S. Cook

**Affiliations:** 1 Department of Sociology, Utrecht University, Utrecht, The Netherlands; 2 Utrecht School of Economics, Utrecht University, Utrecht, The Netherlands; 3 Department of Sociology, Utrecht University, Utrecht, The Netherlands; 4 Department of Sociology, Stanford University, Stanford, California, United States of America; Tianjin University of Technology, CHINA

## Abstract

Despite the popularity of the notion that social cohesion in the form of dense social networks promotes cooperation in Prisoner’s Dilemmas through reputation, very little experimental evidence for this claim exists. We address this issue by testing hypotheses from one of the few rigorous game-theoretic models on this topic, the Raub & Weesie model, in two incentivized lab experiments. In the experiments, 156 subjects played repeated two-person PDs in groups of six. In the “atomized interactions” condition, subjects were only informed about the outcomes of their own interactions, while in the “embedded” condition, subjects were informed about the outcomes of all interactions in their group, allowing for reputation effects. The design of the experiments followed the specification of the RW model as closely as possible. For those aspects of the model that had to be modified to allow practical implementation in an experiment, we present additional analyses that show that these modifications do not affect the predictions. Contrary to expectations, we do not find that cooperation is higher in the embedded condition than in the atomized interaction. Instead, our results are consistent with an interpretation of the RW model that includes random noise, or with learning models of cooperation in networks.

## Introduction

Cooperation is a cornerstone of human societies [1,2]. In many instances of social interaction, people join forces to achieve something they could not have achieved alone. Achieving cooperation, however, is often problematic: actors may face incentives to free-ride on the efforts of others, with the result that cooperation never materializes and the payoff to all actors involved is lower than it would have been, had they cooperated. Consider, for example, two researchers who can collaborate on a project, but are also tempted to let the other do most of the work and focus on their individual projects. This situation is formally captured for two actors in the famous *Prisoner’s Dilemma* (PD). The question as to under which conditions cooperation between rational, selfish actors becomes more likely is one of the major problems of the social sciences, and is also known in sociology as the *problem of social order* [3] or the *problem of social cohesion* [4].

A key finding in this line of research is that cooperation is possible if interactions are repeated [5,6]. However, the assumptions under which this result was initially obtained were rather restricted. Consequently, scholars have searched for additional mechanisms that facilitate the emergence of cooperation.

An important assumption in the ‘baseline’ scenario of repeated interaction is that interactions occur in social isolation. That is, actors interact only with one partner at a time and have no information about interactions in which they are not involved. In reality, however, cooperative relations are often *embedded* in social networks through which information on what happens in one interaction becomes known to third parties [7]. An intuitive and broadly shared view among social scientists is that in such ‘embedded scenarios’ the emergence of cooperation is more likely [8–10], a view supported by much qualitative [11–16] and some quantitative evidence [17,18]. In our example, cooperation in common research projects would be more likely in departments with dense networks, in which information about defections is easily shared among colleagues. This information can impact cooperation in social dilemmas through *reputation effects*. Actors embedded in networks may be more reluctant to defect because word regarding their behavior will spread and lead to sanctions by third parties. In a game-theoretic analysis Raub and Weesie ([19]; the RW model hereafter) show that such reputation effects indeed render conditional cooperation by selfish and rational actors more likely. Moreover, actors may *learn* from previous experiences that cooperation with certain partners is more profitable [20].

## Related Experimental Literature

Among the vast experimental literature on cooperation in the Prisoner’s Dilemma, there are remarkably few studies assessing the effects of network embeddedness. Nevertheless, we identify a number of strands of literature that at least border on our research question. Kollock [21] provides a more general overview of experimental social dilemma research from a sociological perspective; Camerer [22] and Kagel & Roth [23] are good surveys of the broader experimental economics literature.

First, there are studies on the effects of reputation building and communication in the repeated Prisoner’s Dilemma. Building on the seminal theoretical paper by Kreps and Wilson [24], Andreoni and Miller [25] show that the possibility for reputation building in two-person infinitely repeated Prisoner’s Dilemmas increases the likelihood of cooperation, as compared to isolated interaction. Dal Bo [26] and Dal Bo and Frechette [27] show that repeated interaction also increases cooperation in (quasi-)infinitely repeated games, and more so than in infinitely repeated games.

More broadly, experiments tend to show that the possibility of communication, either before or during the game, promotes cooperation, as summarized in meta-analyses by Sally [28] and Balliet [29]. Communication seems particularly effective if it takes place in face-to-face and in larger groups. However, these studies do not consider communication and reputation building in the context of social networks, in the sense that actors receive information via third parties.

A second strand of literature studies the effect of network structure on cooperation. With the growing popularity of both social network analysis and experimental game theory in the past decade, the number of experiments that study strategic interaction in networks in the lab has increased accordingly (see [30] for an older review). Nevertheless, the number of studies that consider cooperation in networks is relatively limited. In almost all cases, these studies focus on *N-*person games, in which, in contrast with our setup, subjects choose one action against all their interaction partners. In the absence of third-party information, ignoring dyadic interaction (in which actors can choose different actions against different partners) in network experiments makes perfect sense, as in that case interactions in different dyads are strategically independent and theoretically not different from isolated encounters. Generally, this research is motivated by evolutionary models such as Nowak and May [31] and others [32,33], which predict that local, structured interaction (as opposed to random interaction) promotes cooperation via imitation of successful partners. Other models predict that certain network structures in particular, such as small-world networks [34], facilitate cooperation. Experimental support for these ideas is limited, however. Kirchkamp and Nagel [35] find no evidence for the use of the “copy-best” strategies assumed by Nowak and May [31], and find that local interaction may even have negative effects under certain conditions. Cassar [36] compares different network structures and finds that while cooperation in clustered networks is higher than in random networks, it is lowest in small-world networks. In experiments with very large networks, however, no evidence of the effects of network structure on cooperation was found [37,38].

A number of recent studies focus on cooperation in dynamic networks. Rand et al. [39] find that both stable and slowly changing social network structures do not improve cooperation; only when subjects are allowed to change interaction partners frequently, cooperation is higher, a finding that is consistent with Riedl and Ule [40]. In contrast, when reputation effects are present in the sense that subjects are informed about the actions of all other subjects, cooperation emerges more consistently [41,42]. These experiments, however, concern *N-*person Prisoner’s Dilemmas in which actors choose a single action against all neighbors, which is strategically different from our setup.

Closer to our research question are studies on the effects of reputation building in networks on trust. Instead of the Prisoner’s Dilemma, these studies rely on the *trust game*, which can be interpreted as a one-sided, sequential version of the Prisoner’s Dilemma. Also in this situation, game-theoretic arguments predict that embeddedness in networks that allow for spreading information leads to more trust [18]. A distinction can be made between *control* effects, which rely on reputation building and forward-looking behavior as assumed by the RW model and *learning* effects, which rely on the use of information on past behavior and do not require actors to worry about their future payoffs. Buskens et al. [43] test these hypotheses in a lab experiment and find evidence for learning effects on trust, but much less for control effects.

The study that comes closest to our study is the experiment by Rapoport et al.[44], which is to our knowledge in fact the only other experiment to explicitly test the RW model. Comparing a condition in which subjects received feedback about actions of other subjects to a condition without such feedback, the authors find that cooperation is higher in the condition with feedback, in line with the theory. However, the analyses do not contain significance tests and do not account for the nested structure of the data. To some extent, our study may be considered a replication of the Rapoport et al. [44] experiment. Details on the differences between the designs are provided below.

Finally, Ahn et al. [45] study reputation effects on dyadic Prisoner’s Dilemmas in a dynamic network context, in the sense that subjects may have the opportunity to choose their interaction partners. They find that in this context, reputation effects enhance cooperation. Besides the dynamic aspect, their setup differs from ours in that information exchange is voluntary and costly which introduces additional strategic considerations into the game. In our setup, following the RW model, we abstract from such voluntary information exchange in order to be able to isolate the effects of information *availability* per se.

## The Raub & Weesie Model

Raub and Weesie [19] formulated their model for reputation effects in social networks in response to Granovetter’s [7] manifest in which he argued for combining the paradigm of rational choice theory with “embeddedness,” that is, explicitly modelling how social structure facilitates cooperative behavior given rational actions of the actors involved.

The model illustrates how the availability of third-party information can improve the possibility for cooperation in the repeated (dyadic) Prisoner’s Dilemma. Key assumptions of the model are:

1. Actors play dyadic infinitely repeated Prisoner’s Dilemmas with multiple partners
2. At each period, exactly one interaction takes place
3. Actors discount future payoffs at a constant rate
4. Payoffs and discount parameters are identical across actors
5. In the *atomized condition* actors observe only the outcomes of their own interactions
6. In the *embedded condition*, actors observe the outcomes of their own interactions and the interactions of all their partners.

Raub and Weesie [19] focus on the conditions under which mutual conditional cooperation can be an equilibrium in this setting. Based on Friedman [46], they derive the necessary and sufficient conditions under which conditional cooperation can be an equilibrium for rational and selfish actors, by showing the equilibrium conditions for *trigger strategies*. These are strategies in which an actor *i* initially cooperates with any other actor *j* and continues to cooperate with *j* as long as *i* does not have any information that *j* defected against *i* or against any other actor than *i*; but actor *i* will defect forever against actor *j* as soon as he observes any defection of *j*.

The conditions under which the mutual use of trigger strategies is a Nash equilibrium of the repeated game can be characterized in terms of the minimal discount parameter that actors need to apply to make mutual conditional cooperation sufficiently profitable. Generally, it can be shown that if the discount parameter is high enough given the payoffs of the game (i.e., actors care enough about long-term payoffs as compared to short-term payoffs), conditional cooperation is a Nash equilibrium in the repeated PD [5,6], although typically not the unique equilibrium. The main result of the analysis by Raub and Weesie [19] is that this minimal value of the discount parameter is lower in the embedded setting than in the atomized condition.

We note that one could implement a slightly stronger version of the trigger strategy implying that an actor stops cooperating completely as soon as he observes any defection by any other actor. We find this stronger version of the trigger strategy less appealing because it implies that actors start defecting with others about whom they have no negative information at all, although this alternative version implies stronger punishment and would therefore also induce more cooperative behavior. This alternative implementation of the trigger strategy would lead to more laborious calculations, but would not change the essence of our hypotheses.

There are two assumptions in the RW model that are problematic for an experimental set-up. First, only one pair of actors interacts in each period. Second, the game is an infinitely (or indefinitely) repeated game. To start with the second issue, it is often impractical if not impossible to implement an indefinitely repeated game in the laboratory, especially if the duration of the game itself cannot be reduced. Indefinitely repeated games might lead to very long repetitions and the subjects would easily realize that they cannot be asked to remain indefinitely in the laboratory. Because of the networks that we implement, we want subjects to interact for some time to allow reputation effects to be established and, thus, the continuation probability should not be too low in our experiment. Therefore, we defer to lengthy finitely repeated games in the experiment. Outside the context of networks, we know from recent experimental evidence [47] that behavior in finitely and infinitely repeated Prisoner’s Dilemmas resemble each other at least until several rounds before the end, although endgame effects in finitely repeated games become a bit more pronounced with experience (see also [26]). Consequently, we base our predictions on results for infinitely repeated games. Still, it is important to realize that similar network effects can also be derived for finitely repeated games with incomplete information (see [48] for an example with Trust Games in networks).

Considering the first problematic assumption, it would be impractical to have only one interaction in a network per period, because it would lengthen the experiment in an unacceptable manner. Given our set-up with a complete network of six subjects, subjects could only make decisions in one out of three rounds and would sometimes even have to wait longer given the randomizations of the relations chosen to interact. Because boredom can seriously affect the behavior of subjects, we changed the set-up so that waiting times were avoided. Fortunately, this assumption is just a convenience assumption in the RW-model that also allows modeling imperfectly embedded interactions in a rather straightforward manner. The assumption can be relaxed in a way that is more practical for a laboratory experiment without changing the main substantive implication. Therefore, we discuss the derivation of the conditions for cooperative equilibria below for the set-up that is also used in the laboratory with the only exception that the game in the laboratory takes 40 periods rather than being infinitely repeated.

**Fig 1:**
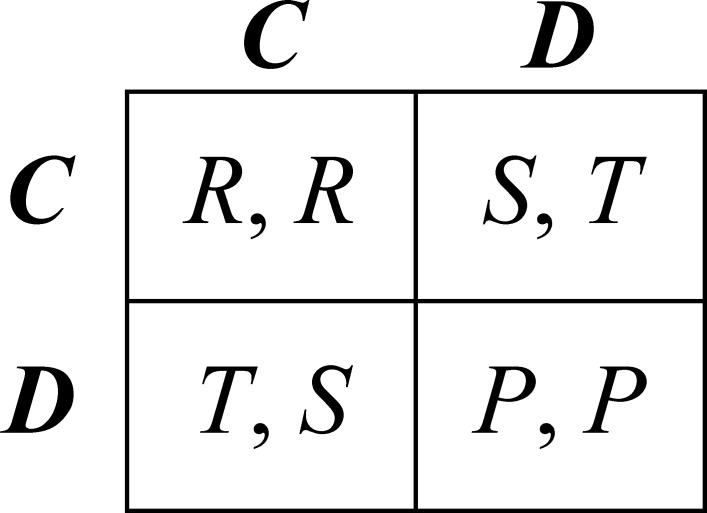
The Prisoner’s Dilemma (*T* > *R* > *P* > *S*).

Assume *n* actors are arranged in a complete network, i.e., every actor can interact with every other actor in the network. Let *m* denote the number of interaction partners actors have in each period *t* of the game. When interaction takes place, agents find themselves in a (symmetric) Prisoner’s Dilemma situation, which is characterized by the payoff matrix in Fig 1. The actors’ possible choices, cooperation (*C*) and defection (*D*) are labelled in the conventional matter. If two actors do not interact in a given period, we assume this relation provides a fixed payoff of *Q*.

Now assume this game is repeated infinitely while payoffs are discounted each round with a parameter β or, equivalently, payoffs are not discounted, but the game is repeated indefinitely with a continuation probability of β. In every period, a random set of relations of the network with *n* actors is chosen (each relation with equal probability) such that each actor interacts with exactly *m* others. We realize that this is only feasible for convenient combinations of *n* and *m*.

We consider now two variants of this game: one in which actors only know the outcomes of their own interactions (atomized) and one in which they know the outcomes of all interactions in the network (embedded). The difference is that in the atomized interactions trigger strategies can only be based on observations in one’s own interactions, while trigger strategies in embedded interactions will also be based on outcomes of interactions between others. More precisely, the trigger strategies imply that, in the atomized encounters, an actor stops cooperating with a partner as soon as this interaction partner defects against the focal actor, while, in embedded encounters, an actor also stops cooperating with a partner as soon as this partner defects against someone else.

As is explained in [19] and [46], we can find the conditions for which conditional cooperation is an equilibrium by deriving the minimal discount parameter for which always cooperating (e.g., ALL-*C* or any other strategy that would play always *C* against trigger) is a best response against the trigger strategy rather than always defecting (ALL-*D*).

The expected future benefits of actor *i* in case he plays ALL-*C* against trigger is the same in both network conditions, because the actor receives *m* times the *R* payoff in every period and (*n*– 1)–*m* times the *Q* payoff for not interacting with some actors in that period. Thus, the expected payoff is given as:

$$EU_{i}(\text{ALL}-C)=\sum_{t=1}^{\infty }\beta^{t-1}(mR+((n-1)-m)Q)=\frac{mR+((n-1)-m)Q}{\left(1-\beta \right)}.$$

The expected future benefits of actor *i* in case he plays ALL-*D* in all future interactions while all his interaction partners use the trigger strategy depends on the embeddedness assumption. In the embedded condition, the actor can defect with *m* others in the first period, but will encounter defection with all actors thereafter because everyone is immediately informed. This leads to the following expected payoff:

$$\begin{array}{l} EU_{i}(\text{ALL}-D|\text{embedded})\\ =mT+((n-1)-m)Q+\sum_{t=2}^{\infty }\beta^{t-1}\left(mP+((n-1)-m)Q\right)=mT+\frac{\beta mP}{1-\beta }+\frac{((n-1)-m)Q}{1-\beta }. \end{array}$$

The calculation for the atomized encounters is a bit more difficult, because not every relation is used at every time point, while in terms of information the relations operate independently. Therefore, lets define π = *m*/(*n*– 1) as the probability that a relation is used to interact at some point *t*. Then, the payoff for this specific relation for the ALL-*D* at some point *t* can have one of three values: (1) with probability 1 – π it equals *Q*, because with this probability the relation is not active; (2) with probability π(1 – π)^*t*–1^ it equals *T*, because this is the probability that the relation was never active from time 1 to *t*– 1 and is first active at time *t*, so the other actor has no information yet and cooperates; (3) with probability π(1 –(1 – π)^*t*–1^), the payoff equals *P*, because this is the probability that the relation is active at time *t* and the actor has already had the opportunity to defect at an earlier point in time. This implies that the expected payoff for playing ALL-*D* in the atomized condition equals:

$$\begin{array}{c} EU_{i}(\text{ALL}-D|\text{atomized})=(n-1)\left(\sum_{t=1}^{\infty }\beta^{t-1}\left((\pi {\left(1-\pi \right)}^{t-1}T+\pi (1-{\left(1-\pi \right)}^{t-1})P+(1-\pi )Q\right)\right)\\ =(n-1)\pi \sum_{t=1}^{\infty }\beta^{t-1}\left(P+{\left(1-\pi \right)}^{t-1}(T-P)\right)+\frac{(n-1)(1-\pi )Q}{1-\beta }\\ =\frac{mP}{1-\beta }+\frac{m(T-P)}{1-\beta (1-\pi )}+\frac{((n-1)-m)Q}{1-\beta } \end{array}$$

For both conditions, we can now derive the threshold value for β for which trigger strategies are an equilibrium by deriving when the expected payoff for ALL-*C* is larger than the expected payoff for ALL-*D*. This implies that for the embedded condition the following should hold:

$$\begin{array}{c} \frac{mR+((n-1)-m)Q}{\left(1-\beta \right)}>mT+\frac{\beta mP}{1-\beta }+\frac{((n-1)-m)Q}{\left(1-\beta \right)}\Leftrightarrow mR>(1-\beta )mT+\beta mP\Leftrightarrow \\ \beta >\frac{T-R}{T-P}=:\beta_{\text{emb}}. \end{array}$$

Thus, the threshold discount parameter for embedded interactions is in our case the well-known condition for conditional cooperation that also holds for repeated interactions in dyads. For atomized interactions it should hold that:

$$\begin{array}{c} \frac{mR+((n-1)-m)Q}{1-\beta }>\frac{mP}{1-\beta }+\frac{m(T-P)}{1-\beta (1-\pi )}+\frac{((n-1)-m)Q}{1-\beta }\Leftrightarrow \\ (1-\beta (1-\pi ))R>(1-\beta (1-\pi ))P+(1-\beta )(T-P)\Leftrightarrow \\ \beta (T-P)-(\beta (1-\pi ))(R-P)>T-R\Leftrightarrow \\ \beta >\frac{T-R}{T-P-(R-P)(1-\pi )}=:\beta_{\text{ato}}>\beta_{\text{emb}}. \end{array}$$

In line with the well-established result of the RW-model, we find thus that the condition for conditional cooperation is more restrictive for atomized interactions than for embedded interactions. The more favorable condition (lower β) for cooperative behavior in embedded interactions comes from the information about the potential misbehavior of a partner getting to the other partners faster than that the other partners interact again with the misbehaving actor. This can also be seen from the formulas, because if π = 1 and everyone would play with everyone else in every period, information spreads equally fast in both conditions and then it holds that β_ato_ = β_emb_. Note also that the payoff *Q* for not interacting with one of the partners has, as expected, no effect on the equilibrium conditions.

For the experiment we implemented the model parameters and assumptions as follows. Payoffs were *T* = 60, *R* = 40, *S* = 0, and *P* = 20, network size *n* was equal to 6, and the number of interactions partners *m* equal to 2. Thus, π = 0.4. This implies that we compare the thresholds ½ = β_emb_ < β_ato_ = 5/7.

Before we formulate our testable hypotheses, it is important to realize again that the thresholds derived are the necessary and sufficient conditions for our trigger strategies to be in equilibrium. But given that these conditions are fulfilled, there are infinitely many other equilibria. For example, all actors playing ALL-*D* is also still an equilibrium as well as many combinations of strategies that mix cooperative and defective behavior. In addition, some of the assumptions of the model will be violated in the laboratory. Not only is the game not infinitely often repeated, it may be the case that actors are often neither rational nor selfish or they do not believe that others are rational and selfish. Because of these complicating factors, it is not reasonable to interpret the results of the theoretic analysis too strictly, but what remains is that under embeddedness the conditions for cooperation are weaker than under atomized interactions. In line with this reasoning, we formulate our hypotheses in a comparative statics manner rather than deterministically predicting specific behaviors under specific conditions.

Directly based on the comparison of the thresholds we hypothesize:

### Hypothesis 1

Average cooperation in a group is higher in embedded interactions than in atomized interactions.

By adding some additional arguments, we can extend this general prediction to predictions concerning the specific phases of the interactions. First, if many cooperative relations turn bad, i.e., despite the fact that cooperative behavior is possible, some defective behavior might occur. This might actually lead to a reversed effect, because in the embedded interactions, information on occasional defections also spreads more easily to other actors. As a result, embeddedness might in such situations decrease cooperation rather than increase it. This argument cannot affect cooperation in the initial interactions. Therefore, because the circumstances for cooperation are also better in embedded interactions at the beginning, we formulate the following hypothesis for initial interactions:

### Hypothesis 2

Average cooperation The likelihood of cooperation in the first interaction of any two actors in a group is larger in embedded interactions than in atomized interactions.

## Experimental Design

To test the hypotheses, we ran two sets of experiments with two conditions each. The two experiments were approved by the IRB of Stanford University (protocol nr 20773) and the IRB of University of California at Berkeley (protocol nr. 2011-06-3374), respectively. Written informed consent was obtained from all participants.

**Fig 2:**
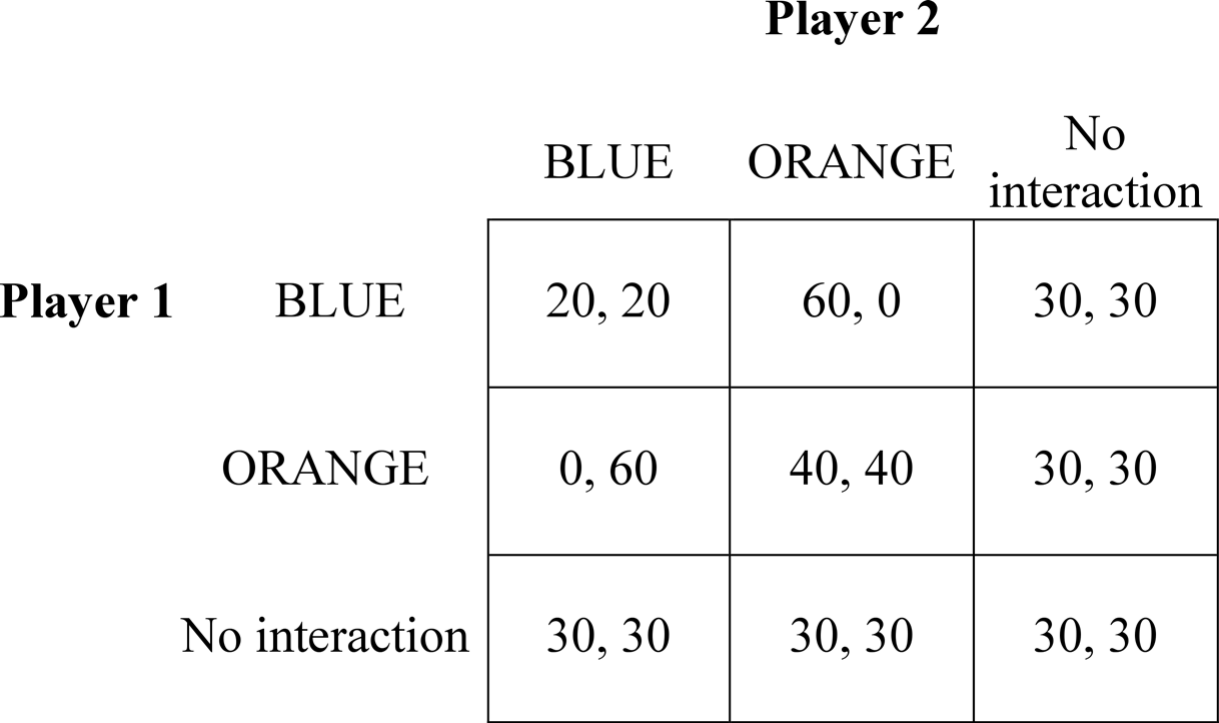
The experimental game.

In the *atomized condition*, subjects interacted in groups of six (note that our labeling of the conditions is inconsistent with Raub and Weesie [19], who use the label “local information” for embedded interaction). Each experimental session consisted of 40 periods. In each period, subjects were randomly matched with two other subjects in their group. They then played a game with every other subject as shown in Fig 2. As Fig 2 shows, subjects received 30 points for every interaction that was not matched in any given period. This implies that in every period, subjects received 3*30 = 90 points, on top of the payoffs from matched interactions, regardless of their choices. The payoff for non-matched interactions was implemented for comparison with a different experiment that is not reported here. Because these payoffs do not in any way depend on the subjects’ choices, they are not expected to influence the results.

If two subjects were matched, they could choose between “BLUE” or “ORANGE,” respectively referring to defection and cooperation in the Prisoner’s Dilemma. If two subjects were not matched, they automatically received the “no interaction” payoff. Thus, in each period, each subject always received the “no interaction” payoff three times, in addition to the payoffs resulting from two Prisoner’s Dilemmas in which they participated. After each period, subjects were informed about the actions of their matched partners and their own payoffs, and about who was matched with whom in the rest of the group. They had access to the history of the outcomes for each period at any moment during the 40 periods of the experiment.

The second condition, which we label the *embedded condition*, was identical to the atomistic condition with the exception that subjects were informed not only about the outcomes of their own interactions, but also about the outcomes of all other interactions.

**Fig 3:**
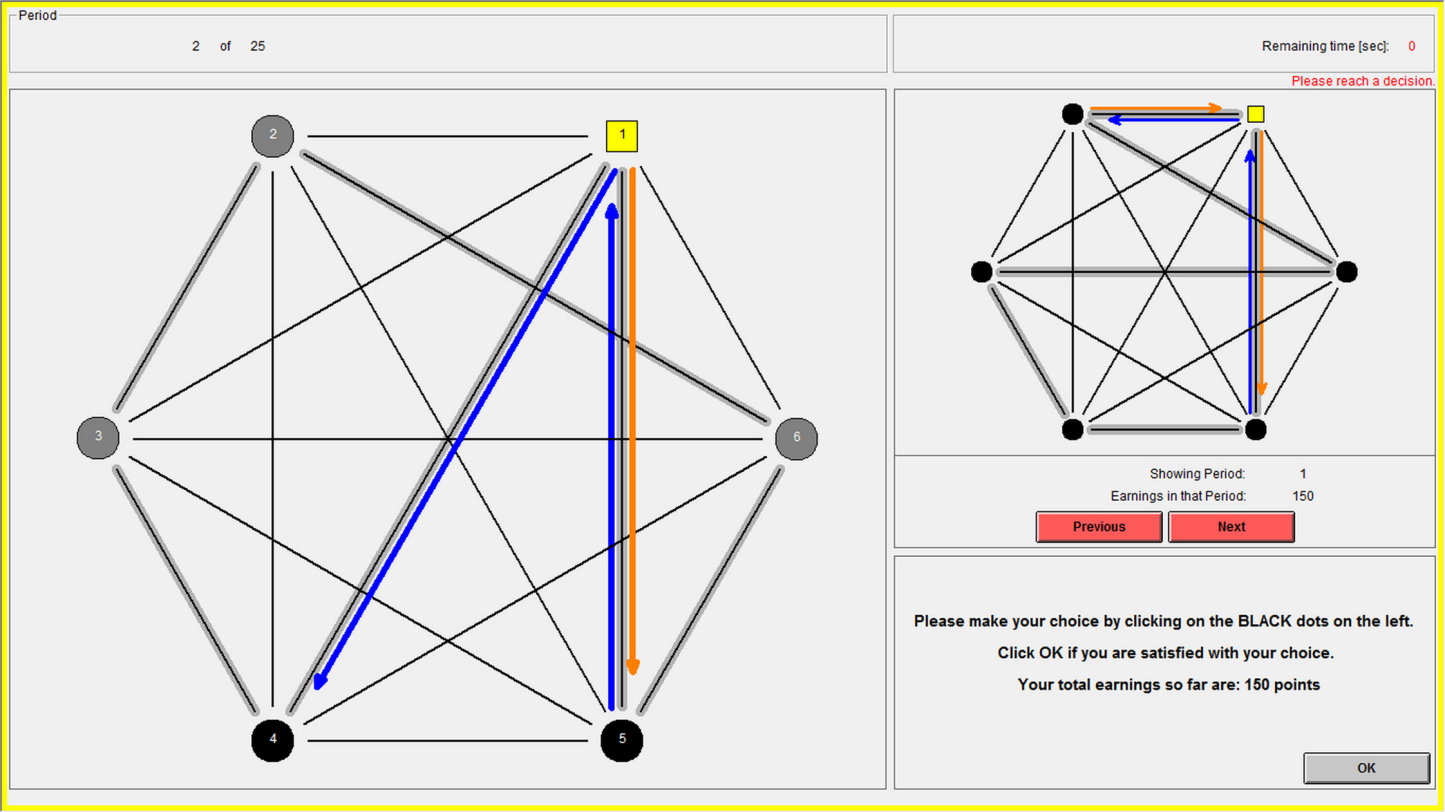
The computer interface: the choice screen of the atomistic condition.

The two conditions were implemented in a computer interface using z-Tree [49]. Fig 3 shows the screen on which subjects made their decisions in the atomistic condition.

The left-hand side of the screen represents the current choice situation. The yellow square represents the focal subject (Ego); the other subjects are represented by circles. The thin black lines between subjects indicate all potential interactions in this group (in the case of the study reported here, these were *all* dyads in the group). The black circles represent the other subjects with whom Ego was actually matched for this period (subjects 4 and 5, in the example). This is indicated by the thick grey lines behind the thin black lines. By observing these thick grey lines, Ego can also learn which other pairs were matched in this period (in the example, these are 2 and 6, 2 and 3, 3 and 4, and 5 and 6).

The choices of the subjects are represented in the interface by arrows: if Ego chooses to play ORANGE against a partner, this is indicated by an orange arrow *from* Ego *to* this partner. Ego can indicate her choice by clicking with the cursor on the circles of the matched partners, which will change the color of the arrow. If Ego interacted before with any of her matched partners, the choices that were made in that previous interaction are already displayed on the screen and Ego can update her own choice as desired. The upper right-hand corner of the screen shows the history of outcomes so far, which Ego can freely browse (using the “next” and “previous” buttons) for reference.

**Fig 4:**
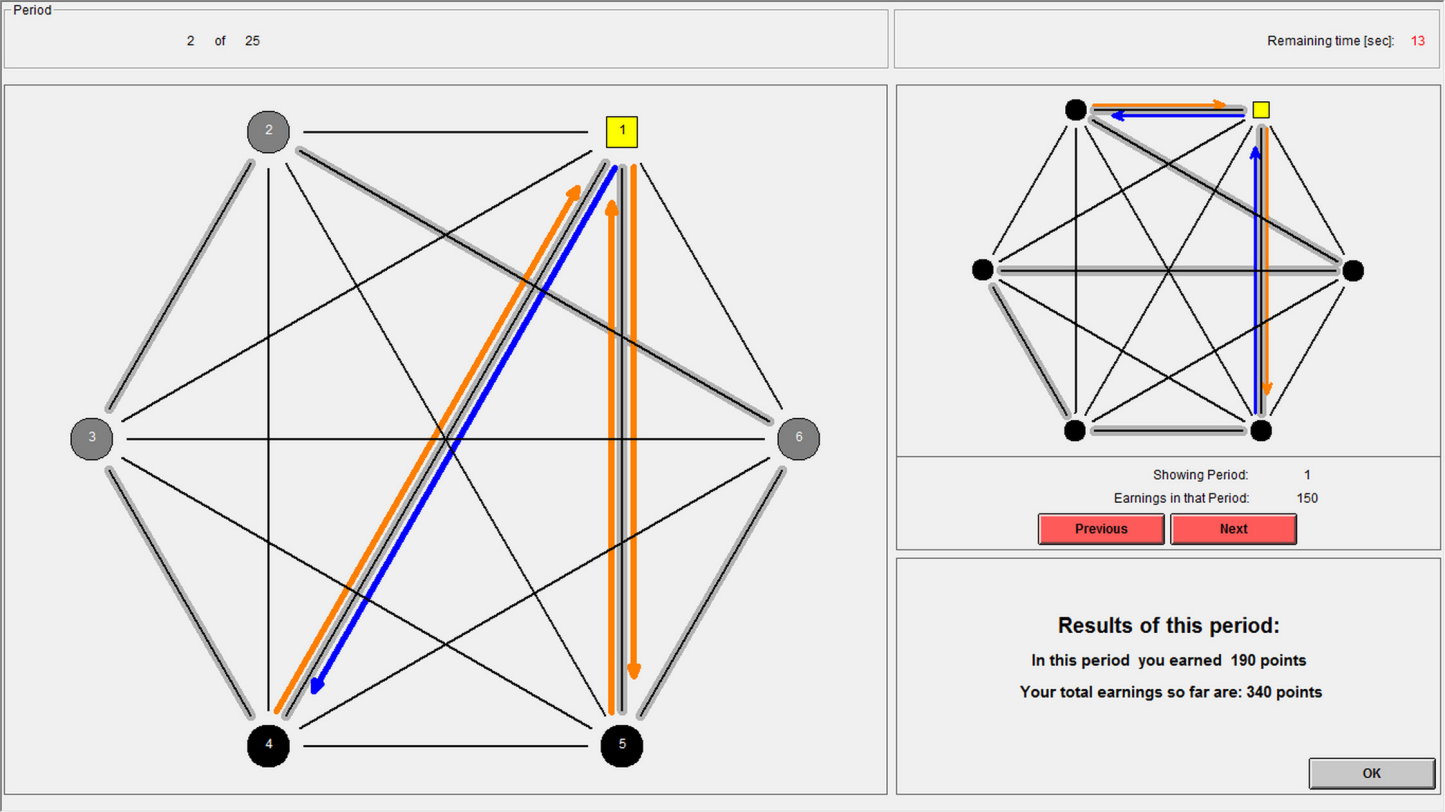
The computer interface: the results screen of the atomistic condition.

When Ego is satisfied with her choice, she clicks “OK,” which brings up the results screen shown in Fig 4. This screen shows the actions of Ego and her interaction partners and reports Ego’s payoffs. In this example, Ego earned 40 points from the interaction with subject 5, 60 from the interaction with subject 4, and three times 30 for the other subjects with whom she did not interact, totaling 190 points. As in the choice screen, the upper right-hand corner of the screen provides the history of previous outcomes for reference.

**Fig 5:**
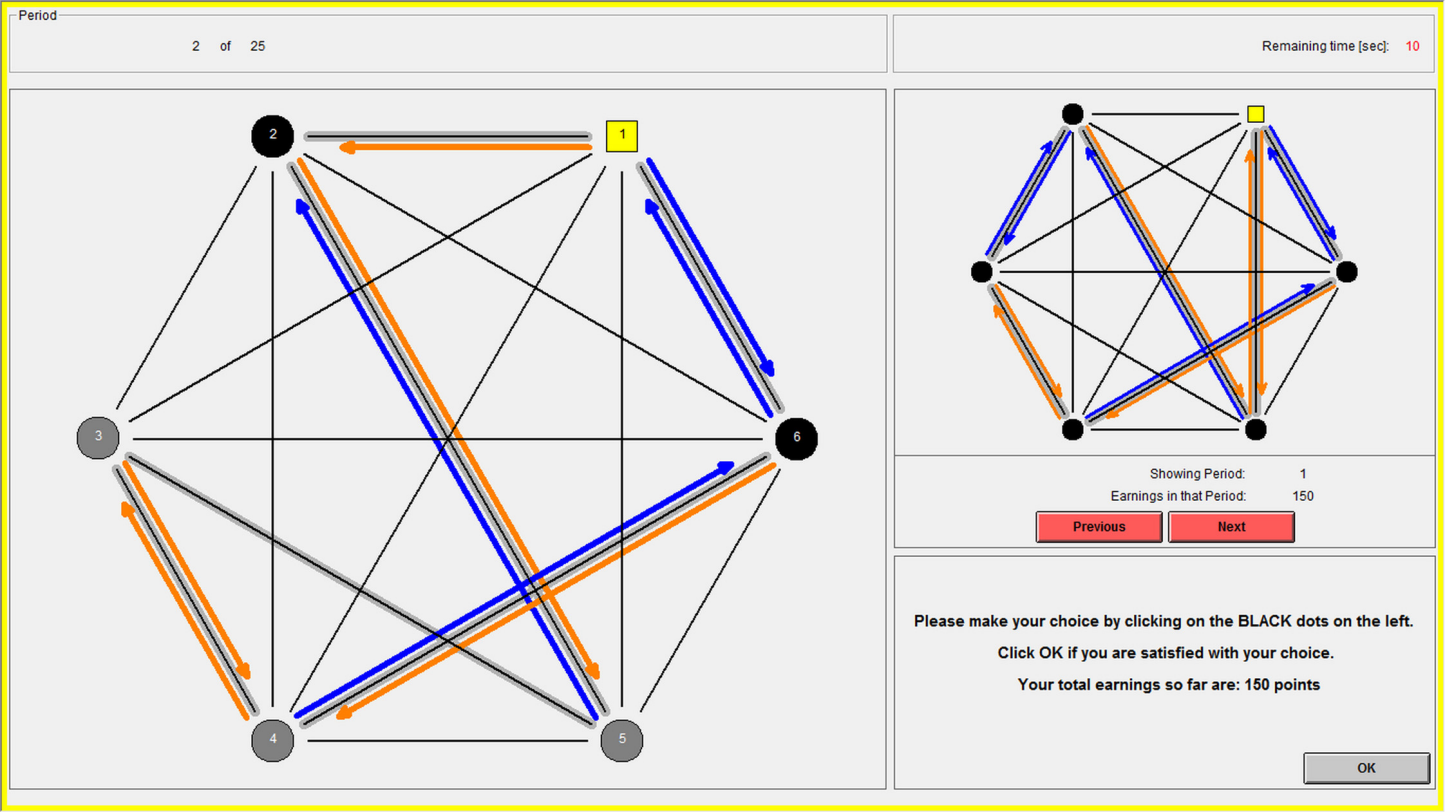
The computer interface: the results screen of the embeddedness condition.

The interface of the embeddedness condition differs from the above only to the extent that outcomes of all other interactions are also displayed, as illustrated by Fig 5, which shows the results screen from the embeddedness condition. Here, arrows are not only displayed for Ego’s own interactions, but also for all other interactions that took place in that period.

Subjects were instructed about the details of the game and the interface through a set of written instructions, which they had available throughout the experiment for reference. Before the 40 periods of the experiment began, subjects played five “practice periods” to familiarize themselves with the interface and the game. After the 40^th^ period, subjects were shown an overview of the total number of points they had earned.

### Experiment 1

#### Setup

In the first set of experiments, we ran 13 sessions with a total of 14 6-person groups, which implies that we ran two groups simultaneously only once. The sessions took place in the experimental laboratory on the campus of a private university in the US during the spring of 2011. The experiment involved a total of 84 subjects of whom 44% were female and 81% were born in the US. The average age was 21.2, and almost all of the subjects were undergraduate students. A typical session lasted about 45 minutes. Each session used the procedure described above. Of the 14 groups, seven groups were placed in the atomistic condition and the other seven in the embeddedness condition.

#### Results

**Fig 6:**
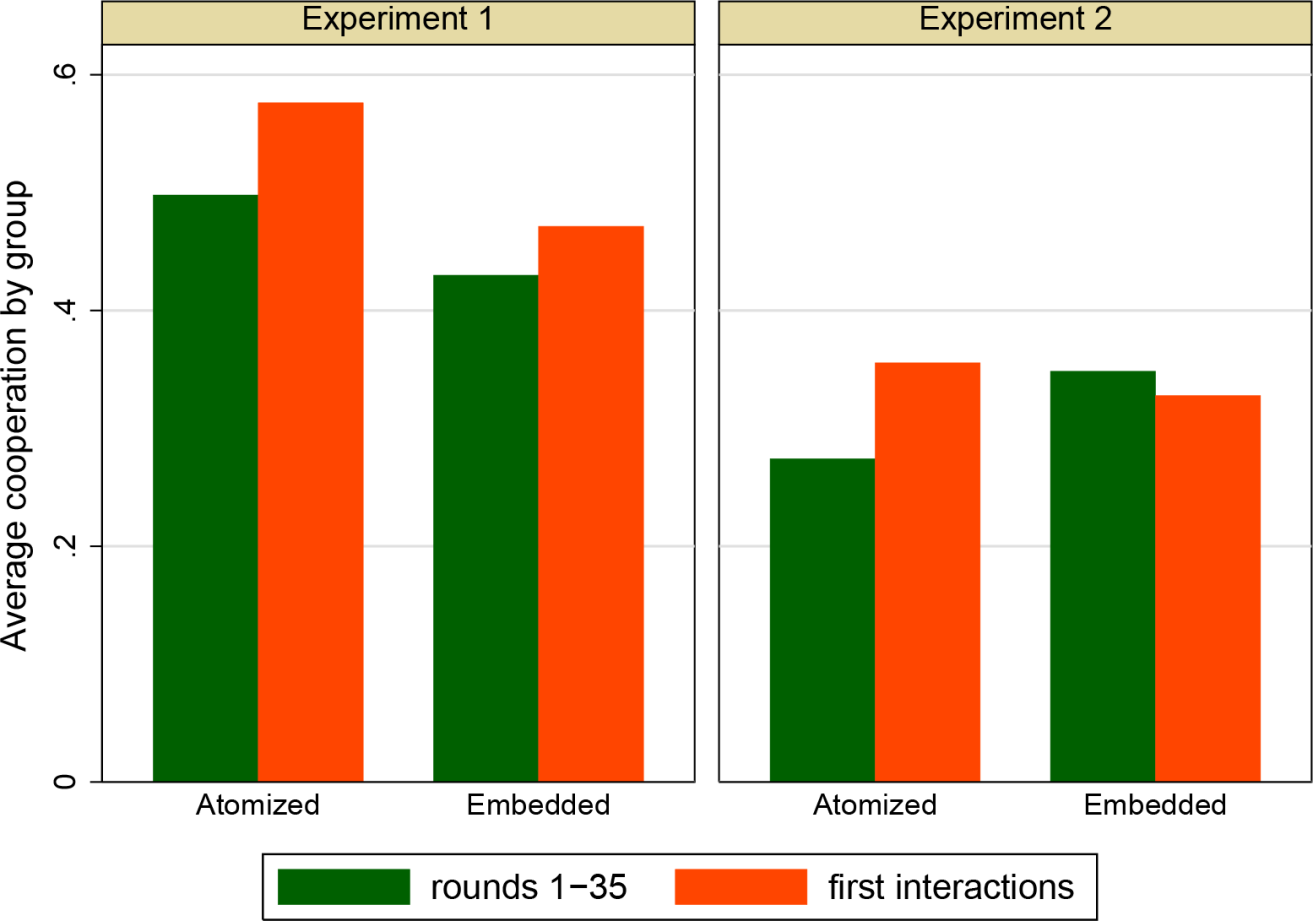
Average levels of cooperation per group, by location (*N =* 26).

The left hand panel of Fig 6 shows the main results with regard to cooperation levels. As is common in the literature, we disregard the final five rounds of the game, as “end game effects” are likely to bias the results in those rounds. In contrast with the prediction in Hypothesis 1, we do not find that overall cooperation levels are higher in the embeddedness condition. Indeed, as the figure shows, cooperation is somewhat *lower* in the embeddedness condition, although this difference is not statistically significant according to a Mann-Whitney test (*N* = 14, *p* = .28).

Hypothesis 2 predicts that cooperation in the first interactions of each pair is higher in the embeddedness condition. This hypothesis is also rejected by the data: again, cooperation levels are *lower* in the embeddedness condition (but not significantly so; *N* = 14, *p* = .22).

### Experiment 2

A possible limitation of the first experiment was that, because we had mostly one group at a time in the lab, anonymity among the subjects was not optimal. This, in combination with the impossibility to reshuffle the groups between the practice periods, might have dampened the hypothesized effect of network embeddedness. Given these possible confounds, we ran a second set of sessions at a different location (a lab at the campus of a public university in the US) where we were assured of having several groups scheduled at the same time. These sessions were conducted in August 2011 and September 2012.

#### Setup

For Experiment 2, we ran 6 sessions with two groups in each session, resulting in 12 groups with a total of 72 subjects. Of these, 60% were female and 60% were born in the US. The average age was 20.3, and again almost all of the subjects were undergraduate students. Because we now had two groups in each session, groups were reshuffled between the practice periods and the actual data collection periods. Apart from that, the procedure was identical to the procedure in Experiment 1. The 12 groups were equally divided into the two experimental conditions (atomized and embedded).

#### Results

The right hand panel of Fig 6 shows the main results of Experiment 2. In contrast with Experiment 1, we observe somewhat higher levels of cooperation in the embeddedness condition. This result, however, is not statistically significant (*N =* 12, *p* = .2), so we again find no support for Hypothesis 1.

While one might attribute this null finding to a lack of statistical power (with a larger number of groups, the result might become significant), we argue that our null finding is unlikely to be a consequence of lack of statistical power for the following two reasons:

First, from a theoretical point of view, if one would like to interpret the positive effect related to Hypothesis 1 as tentative support for the RW model, this would at least require that we would also need tentative support for Hypothesis 2, which predicts higher cooperation in initial interactions in the embeddedness condition than in the atomistic condition. Fig 6 again shows that we do not find this. As in Experiment 1, cooperation levels are, if anything, lower rather than higher in initial interactions in the embeddedness condition (*N* = 12, *p* = .62). This results show that, regardless of statistical significance, our results are not consistent with the theory.

Second, we conducted a power analysis to investigate whether our sample size is adequate to identify the hypothesized effect. Because our hypotheses do not predict a precise effect size, we rely on earlier empirical results as a reasonable guideline for the expected effect size. In our case, the closest other experiment is the one by Rapoport et al. [44], who found a difference in average cooperation rate of about 0.19 between their atomistic and embedded condition (as can be reconstructed from their figures; the mean difference is not actually reported). As Rapoport et al. [44] do not report the variance, we assume a standard deviation of .1 in both treatments, which approximates the average standard deviation in our Experiment 2. A standard power analysis for t-tests shows that with this effect size, a sample size of five would already be sufficient to expect to observe the effect with more than 80% likelihood. The Mann-Whitney test that we use might be slightly more conservative, but this result provides some confidence that our null finding is not a result of a lack of power. Finally, we point out that our sample size is not unusually small as compared to other network experiments in the literature (e.g., [41]).

If we combine the data of both experiments, the results are not much different. As may be inferred from Fig 6, because the effects of embeddedness on overall cooperation (rounds 1–35) are in the opposite directions in both experiments, we hardly see any difference in overall cooperation if we pool the data (*N =* 26, *p =* .82). The effects on cooperation in initial interactions are consistent across the two experiments (contradicting Hypothesis 2), and the difference is still not significant in the pooled dataset (*N* = 26, *p =* .40).

## Alternative Explanations

The model developed by Raub and Weesie [19] relies on a number of rather strong game-theoretic assumptions. First, the model assumes that there is no noise, in the sense that actors do not make random mistakes and that there are no external forces influencing the results of actors’ decisions. The use of trigger strategies suggests, however, that the predictions of the model are highly sensitive to such noise: if one actor defects, even if by accident, all other actors would retaliate by defection and cooperation would break down completely. Reputation effects in this case cause defection to quickly spread through the population, while the consequences of random mistakes would be limited to dyads in atomized interactions. The presence of reputation effects would thus, with higher levels of noise, lead to *less* cooperation, or at least to more *variance* in cooperation levels across groups.

A second strong assumption is that actors are perfectly rational, and in particular that they apply forward-looking reasoning in their decision making. Research on reputation effects in trust games, which arguably represent a simpler social dilemma, has shown that this assumption is problematic [43]. Learning models offer one way to relax this strong assumption. In such models, actors do not maximize their utility over all future periods, but instead rely on information from past interactions to maximize utility in the near future [50]. A particular learning model for cooperation in networks with reputation effects has been studied by Corten and Cook [51]. Using simulations, they show that the presence of reputation effects does not necessarily lead to more cooperation, but instead leads to more variance in cooperation levels between groups.

The two alternative perspectives above thus, on the one hand, provide explanations for the absence of reputation effects and, on the other hand, offer a new prediction: that the variance of cooperation levels across groups is higher in the reputation condition. Note that, given the nature of these explanations, this is a prediction about the *final periods* of the repeated game, rather than all periods or even the first period, which was our focus before.

We consider this alternative hypothesis using Table 1, which shows the means and standard deviations of group cooperation in periods 30–35. The table provides some mild support for the idea that the variance in cooperation is larger in the presence of reputation effects, in particular in the second experiment. Closer analysis reveals that in the first experiment the larger variance is caused by a single outlier. A variance comparison test confirms that the standard deviation is larger in the embedded condition than in the atomistic condition for experiment 2 (*p* = .05), but not for experiment 1 (*p* = .21).

**Table 1:** means and standard deviations of cooperation levels, periods 30–35. Unit of analysis is the group **(*N =* 26).**

	Condition	
	Embedded	Atomistic
**Experiment 1**	0.506	0.502
	(0.131)	(0.187)
**Experiment 2**	0.278	0.354
	(0.060)	(0.133)*

* *p* <0.1.

## Conclusions

In this article we tested the widespread notion that social cohesion promotes cooperation in social dilemma situations through reputation building in social networks. To derive specific hypotheses, we relied on the game-theoretic model by Raub and Weesie [19] showing that the conditions for mutual conditional cooperation in the repeated Prisoner’s Dilemma become less restrictive if actors are embedded in a social network that allows the diffusion of information about interactions other than their own. While there is some experimental empirical support for this prediction [44], experimental tests of this much-cited model are very rare.

We conducted a series of controlled laboratory experiments to test two hypotheses: 1) that average cooperation is higher in groups in which interaction is embedded in social networks as compared to atomized interaction, and 2) that average cooperation *in the first interactions of the game* is higher in embedded groups than in atomized groups. Our experiments were designed to approximate the conditions assumed in the model by Raub and Weesie [19] as closely as possible. The experiments, while using the same design, were conducted at different locations at different times, prompting us to treat them as separate experiments.

The results of the experiments do not lend support to either of the hypotheses. That is, in both experiments, we find no significant difference between the embedded and atomized conditions (H1). In Experiment 2, the effect of embeddedness was in the expected direction, however, suggesting that we might find support for Hypothesis 2 with more observations. The results on the second hypothesis, however, are not consistent with this interpretation. In both experiments, average cooperation in initial interactions is *lower* rather than higher (but not significantly so). Thus, even if we observe higher average cooperation in the embedded condition in the second experiment, this is not because subjects cooperate conditionally from the beginning of the game. Rather, the results seem to suggest that subjects learn to cooperate over time. Indeed, further analyses provide some modest support for learning models of cooperation in networks.

Why do we find no support for the RW-model, while Rapoport et al. [44] indeed find higher cooperation rates in the embedded condition, both on average and in the first round of the game? While it is difficult to pinpoint a clear cause, we do note a number of differences with their study. While we posed some questions related to the statistical significance of the results of Rapoport et al., let us here assume their differences are statistically significant. First, our setup is slightly more complicated than theirs in the sense that in our design, subjects always play two interactions per round, while in their study [44], subjects played only one interaction per round.

Second, our design is also more complicated in the sense that subjects received more information about previous interactions of their interaction partners. In Rapoport et al. [44], subjects were only informed about the previous actions of their interaction partners in other interactions, while in our design, subjects in the embedded condition were informed about the actions not only of their interaction partners, but also the actions *of the interaction partners of their interaction partners*. Thus, in our setup, subjects may distinguish defection by their interaction partners that is retaliation against an earlier defection from “spontaneous” defection. While it is not trivial to derive implications of this ability for overall cooperation rates, we may speculate that it dampens the effect of embeddedness because defection may be less severely punished.

The increased number of interactions and larger amount of information available potentially also increase the influence of random noise in the process. As we have argued above, if actors indeed use trigger strategies as implied by the RW model, reputation effects might even drive down cooperation in the presence of random mistakes by the actors. Our additional analyses show that our results are, to some extent, consistent with this interpretation.

Another speculative explanation for the differences between our findings and those reported by Rapoport et al. [44] is that the added complexity of our design inhibits embeddedness effects, simply because subjects are unable to process the information needed for the embeddedness effect to work. The implication of this would be that embeddedness effects, as predicted by the RW model, are limited to very simple settings, in which subjects have very little information to evaluate.

In a broader perspective, our results suggest that the widely observed association between social cohesion and cooperation might not be explained by reputation mechanisms that make conditional cooperation more attractive for forward-looking actors, as theorized by Raub and Weesie [19]. To conclude the paper, we briefly comment on alternative explanations of this association.

First, it may be that social cohesion fosters cooperation by other mechanisms than reputation building among forward-looking actors. Such alternative mechanisms may involve learning by boundedly rational, backward looking actors [20] or adaptive behavior [31], although experimental support for the latter is also limited [37]. Still, if interactions in more cooperative groups are more likely to be sustained, learning might have a biased effect towards cooperation, although embeddedness can also inhibit cooperation because information about defection is diffused faster as well.

A second possibility is that social cohesion does not generally foster cooperation, but that the causal relation is in the opposite direction: high cooperation rates lead to social cohesion. Studies suggesting that the possibility of partner choice promotes cooperation [39,40], allowing cooperators to form clusters, seem consistent with this interpretation. Further research will have to examine the role of reputation mechanisms in such processes, because in the experiments reported here, subjects did not have the possibility to alter their relationships and thus could not avoid interactions with subjects who had been uncooperative. First theoretical attempts indicate that this role may be ambivalent [51], but It is clear that further experimental research is needed to disentangle the effects of partner choice and reputation effects on cooperation in a dynamic context.

## References

[1] Ostrom E. Governing the commons: The evolution of institutions for collective action. Cambridge, MA: Cambridge University Press; 1990.

[2] Pennisi E. How Did Cooperative Behavior Evolve? Science (80-). 2005;309: 93. 10.1126/science.309.5731.93 15994539

[3] Parsons T. The Structure of Social Action Volume I: Marshall, Pareto, Durkheim. New York: Free Press; 1937.

[4] Ultee W, Arts W, Flap H. Sociologie; Vragen, Uitspraken, Bevindingen. Groningen: Wolters-Noordhof; 1996.

[5] Axelrod R. The Evolution of Cooperation. New York: Basic Books; 1984.

[6] Taylor M. Anarchy and Cooperation. London: Wiley; 1976.

[7] Granovetter MS. Economic Action and Social Structure: The Problem of Embeddedness. Am J Sociol. 1985;91: 481–510.

[8] Homans GC. The Human Group. London: Routledge; 1951.

[9] Coleman JS. Foundations of Social Theory. Cambridge, MA: Belknap; 1990.

[10] Voss T. Game-Theoretical Perspectives on the Emergence of Social Norms In: Hechter M, Opp K-D, editors. Social Norms. New York: Russell Sage Foundation; 2001 pp. 105–136.

[11] Macaulay S. Non-Contractual Relations in Business: A Preliminary Study. Am Sociol Rev. 1963;28: 55–67.

[12] Greif A. Reputation and Coalitions in Medieval Trade: Evidence on the Maghribi Traders. J Econ Hist. 1989;49: 857–882.

[13] Greif A. Cultural Beliefs and the Organization of Society: A Historical and Theoretical Reflection on Collectivist and Individualist Societies. J Polit Econ. 1994;102: 912–950.

[14] Ellickson RC. Order without Law How Neighbors Settle Disputes. Cambridge, MA: Harvard University Press; 1991.

[15] Uzzi B. The Sources and Consequences of Embeddedness for the Economic Performance of Organizations: The Network Effect. Am Sociol Rev. 1996;61: 674–698.

[16] Uzzi B. Social Structure and Competition in Interfirm Networks: The Paradox of Embeddedness. Adm Sci Q. 1997;42: 35–67.

[17] Burt RS, Knez M. Trust and Third-Party Gossip In: Kramer RM, Tyler TR, editors. Trust in Organizations: Frontiers of Theory and Research. Thousand Oaks, CA: Sage; 1996 pp. 68–89.

[18] Buskens V. Social Networks and Trust. Boston, MA: Kluwer Academic Publishers; 2002.

[19] Raub W, Weesie J. Reputation and Efficiency in Social Interactions: An Example of Network Effects. Am J Sociol. 1990;96: 626–654.

[20] Buskens VW, Raub W. Embedded Trust: Control and Learning In: Thye SR, Lawler EJ, editors. Group Cohesion, Trust and Solidarity. Amsterdam: Elsevier Science; 2002 pp. 167–202.

[21] Kollock P. Social Dilemmas: The Anatomy of Cooperation. Annu Rev Sociol. 1998;24: 183–214.

[22] Camerer CF. Behavioral Game Theory: Experiments in Strategic Interaction. Princeton, NJ: Princeton University Press; 2003.

[23] Kagel JH, Roth AE, editors. The Handbook of Experimental Economics. Princeton, NJ: Princeton University Press; 1995.

[24] Kreps DM, Wilson R. Reputation and Imperfect Information. J Econ Theory. 1982;27: 253–279.

[25] Andreoni J, Miller J. Rational cooperation in the finitely repeated prisoner’s dilemma: Experimental evidence. Econ J. 1993.

[26] Bó PD. Cooperation under the Shadow of the Future: Experimental Evidence from Infinitely Repeated Games. Am Econ Rev. American Economic Association; 2005;95: 1591–1604. 10.1257/000282805775014434

[27] Dal Bó P, Fréchette GR. The Evolution of Cooperation in Infinitely Repeated Games: Experimental Evidence. Am Econ Rev. American Economic Association; 2011;101: 411–429. 10.1257/aer.101.1.411

[28] Sally D. Conversation and Cooperation in Social Dilemmas: A Meta-Analysis of Experiments from 1958 to 1992. Ration Soc. 1995;7: 58–92. 10.1177/1043463195007001004

[29] Balliet D. Communication and Cooperation in Social Dilemmas: A Meta-Analytic Review. J Conflict Resolut. 2009;54: 39–57. 10.1177/0022002709352443

[30] Kosfeld M. Economic Networks in the Laboratory: A Survey. Rev Netw Econ. 2004;3: 20–41.

[31] Nowak M, May R. Evolutionary games and spatial chaos. Nature. 1992.

[32] Wang J, Xia C, Wang Y, Ding S, Sun J. Spatial prisoner’s dilemma games with increasing size of the interaction neighborhood on regular lattices. Chinese Sci Bull. 2012;57: 724–728. 10.1007/s11434-011-4890-4

[33] Ding S, Wang J, Ruan S, Xia C. Inferring to individual diversity promotes the cooperation in the spatial prisoner’s dilemma game. Chaos, Solitons & Fractals. 2015;71: 91–99. 10.1016/j.chaos.2014.12.014

[34] Watts DJ, Strogatz SH. Collective Dynamics of “Small World” Networks. Nature. 1998;393: 440–442. 9623998 10.1038/30918

[35] Kirchkamp O, Nagel R. Naive learning and cooperation in network experiments. Games Econ Behav. 2007;58: 269–292. 10.1016/j.geb.2006.04.002

[36] Cassar A. Coordination and Cooperation in Local, Random and Small World Networks: Experimental Evidence. Games Econ Behav. 2007;58: 209–230.

[37] Gracia-Lázaro C, Ferrer A, Ruiz G, Tarancón A, Cuesta JA, Sánchez A, et al Heterogeneous networks do not promote cooperation when humans play a Prisoner’s Dilemma. Proc Natl Acad Sci U S A. 2012;109: 12922–6. 10.1073/pnas.1206681109 22773811 PMC3420198

[38] Grujić J, Fosco C, Araujo L, Cuesta JA, Sánchez A. Social experiments in the mesoscale: humans playing a spatial prisoner’s dilemma. PLoS One. Public Library of Science; 2010;5: e13749 10.1371/journal.pone.0013749 21103058 PMC2980480

[39] Rand DG, Arbesman S, Christakis NA. Dynamic social networks promote cooperation in experiments with humans. Proc Natl Acad Sci U S A. 2011;108: 19193–8. 10.1073/pnas.1108243108 22084103 PMC3228461

[40] Riedl A, Ule A. Exclusion and cooperation in social network experiments. Unpubl Pap CREED, Univ …. 2002.

[41] Gallo E, Yan C. The effects of reputational and social knowledge on cooperation. Proc Natl Acad Sci. 2015;112: 201415883 10.1073/pnas.1415883112 PMC4378402 25775544

[42] Cuesta JA, Gracia-Lázaro C, Ferrer A, Moreno Y, Sánchez A. Reputation drives cooperative behaviour and network formation in human groups. Sci Rep. Nature Publishing Group; 2015;5: 7843 10.1038/srep07843 25598347 PMC4297950

[43] Buskens V, Raub W, van der Veer J. Trust in triads: An experimental study. Soc Networks. 2010;32: 301–312.

[44] Rapoport A, Diekmann A, Franzen A. Experiments with Social Traps IV: Reputation Effects in the Evolution of Cooperation. Ration Soc. 1995;7: 431–441.

[45] Ahn TK, Esarey J, Scholz JT. Reputation and Cooperation in Voluntary Exchanges: Comparing Local and Central Institutions. J Polit. 2009;71: 398–413.

[46] Friedman J. Oligopoly and the Theory of Games. New York. Amsterdam: North Holland; 1977.

[47] Normann H-T, Wallace B. The impact of the termination rule on cooperation in a prisoner’s dilemma experiment. Int J Game Theory. 2012;41: 707–718.

[48] Buskens V. Trust in triads: effects of exit, control, and learning. Games Econ Behav. 2003;42: 235–252. 10.1016/S0899-8256(02)00563-8

[49] Fischbacher U. Z-Tree: Zurich Toolbox for Readymade Economic Experiments—Experimenter’s Manual. J Exp Econ. 2007;10: 171–178.

[50] Macy MW, Flache A. Learning Dynamics in Social Dilemmas. Proc Natl Acad Sci. 2002;99: 7229–7236. 12011402 10.1073/pnas.092080099 PMC128590

[51] Corten R, Cook KS. Cooperation and Reputation in Dynamic Networks. In: Paolucci M, editor. Proceedings of the First International Conference on Reputation: Theory and Technology—ICORE 09. Gargonza, Italy; 2009. pp. 20–34.

